# Acquisition of regeneration competence in adult fibroblasts is accompanied by upregulation of arginine methyltransferase PRMT8

**DOI:** 10.1186/1756-8935-6-S1-P132

**Published:** 2013-04-08

**Authors:** Sarah Runqe, Tanja Dominko

**Affiliations:** 1 Department of Biology and Biotechnology, Worcester Polytechnic Institute, Worcester, MA, USA; 2 Bioengineering Institute, Worcester Polytechnic Institute, Worcester, MA, USA

## Background

Regardless of whether cancers arise as a consequence of genetic or epigenetic changes, the factors that control the balance between replicative senescence and cancerous self-renewal are of much interest as potential therapeutic targets. However, the molecular mechanisms that regulate this perfect balance are not well understood.

## Materials and methods

To better study this regulatory mechanism, we have developed a model system *in vitro* which allows for increase in TERT levels leading to increased proliferative potential of the cells and increased time to senescence, while at the same time the cells remain non-tumorigenic when injected into SCID mice [1]. This novel phenotype is also accompanied by induction of regeneration competence as demonstrated by significant reduction of collagen deposition in a mouse skeletal wound [2], leading us to coin the term induced regeneration competent (iRC) cells. Understanding the mechanism of cell plasticity in the context of a defined environment will offer molecular tools for designing of regenerative instead of symptomatic treatment strategies.

## Results

Our preliminary data shows an almost 14 fold transcriptional increase in PRMT8 in iRC cells displaying nuclear localization. Since PRMT8’s debut in 2005, little has been published about the function of this enzyme. However, a variety of biological roles for PRMT family members are being uncovered indicating potential regulatory mechanisms for arginine methylation in cellular senescence.

## Conclusions

Because of the above, we hypothesize that PRMT8 is involved in increased proliferation of iRC cells by direct or indirect regulation of TERT expression and elucidation of this pathway will uncover therapeutic targets for regenerative medicine and cancer research.

## References

[B1] Page RL Ambady S Holmes WF Vilner L Kole D Kashpur O Huntress V Vojtic I Whitton H Dominko T Induction of Stem Cell Gene Expression in Adult Human Fibroblasts without Transgenes Cloning and Stem Cells 2009 11 417 426 10.1089/clo.2009.0015 19622035 PMC2925031

[B2] Page RL Malcuit C Vilner L Vojtic I Shaw S Hedblom E Hu J Pins GD Rolle MW Dominko T Restoration of Skeletal Muscle Defects with Adult Human Cells Delivered on Fibrin Microthreads Tissue Engineering Part A 2011 17 2629 2640 10.1089/ten.tea.2011.0024 21699414

